# Association between heme oxygenase-1 and hyperlipidemia in pre-diabetic patients: a cross-sectional study

**DOI:** 10.3389/fendo.2024.1380163

**Published:** 2024-05-23

**Authors:** Shujin Fan, Yulin Yang, Xiaoyu Li, Jing Liu, Yue Qiu, Li Yan, Meng Ren

**Affiliations:** ^1^ Department of Endocrinology, Sun Yat-Sen Memorial Hospital, Sun Yat-sen University, Guangzhou, China; ^2^ Guangdong Clinical Research Center for Metabolic Diseases, Guangzhou Key Laboratory for Metabolic Diseases, Guangzhou, China; ^3^ Department of Gastroenterology, The First People’s Hospital of Foshan, Foshan, China

**Keywords:** hyperlipidemia, heme oxygenase-1, pre-diabetes, cross-sectional study, association

## Abstract

**Background:**

Although the importance and benefit of heme oxygenase-1 (HO-1) in diabetes rodent models has been known, the contribution of HO-1 in the pre-diabetic patients with hyperlipidemia risk still remains unclear. This cross-sectional study aims to evaluate whether HO-1 is associated with hyperlipidemia in pre-diabetes.

**Methods:**

Serum level of HO-1 was detected using commercially available ELISA kit among 1,425 participants aged 49.3–63.9 with pre-diabetes in a multicenter Risk Evaluation of cAncers in Chinese diabeTic Individuals: A lONgitudinal (REACTION) prospective observational study. Levels of total cholesterol (TC) and triglyceride (TG) were measured and used to defined hyperlipidemia. The association between HO-1 and hyperlipidemia was explored in different subgroups.

**Result:**

The level of HO-1 in pre-diabetic patients with hyperlipidemia (181.72 ± 309.57 pg/ml) was obviously lower than that in pre-diabetic patients without hyperlipidemia (322.95 ± 456.37 pg/ml). High level of HO-1 [(210.18,1,746.18) pg/ml] was negatively associated with hyperlipidemia (OR, 0.60; 95% CI, 0.37–0.97; p = 0.0367) after we adjusted potential confounding factors. In subgroup analysis, high level of HO-1 was negatively associated with hyperlipidemia in overweight pre-diabetic patients (OR, 0.50; 95% CI, 0.3–0.9; p = 0.034), especially in overweight women (OR, 0.42; 95% CI, 0.21–0.84; p = 0.014).

**Conclusions:**

In conclusion, elevated HO-1 level was negatively associated with risk of hyperlipidemia in overweight pre-diabetic patients, especially in female ones. Our findings provide information on the exploratory study of the mechanism of HO-1 in hyperlipidemia, while also suggesting that its mechanism may be influenced by body weight and gender.

## Introduction

The incidence of hyperlipidemia is increasing gradually worldwide (1). According to The China Health and Nutrition Survey in 2012, the proportion of men and women whose total TC levels abnormally increase was 4.7% and 5.1%, respectively, and the men and women whose TG ≥2.26 mmol/L accounted for 16.7% and 9.8%, respectively (2). The characteristics of hyperlipidemia include elevated total cholesterol (TC, more than 200 mg/dL) with or without increased triglycerides (TG, more than 200 mg/dL), low-density lipoprotein cholesterol (LDL-C, more than 100 mg/dL) and decreased high-density lipoprotein cholesterol (HDL-C, less than 60 mg/dL) levels, but these parameters do not always appear simultaneously (3–5). As a common and persistent metabolic disorder, hyperlipidemia tends to cause hyperglycemia, overweightand narrowing of blood vessels (6). It has been reported that elevated blood lipid levels are associated with diabetes (7) and obesity (8). In the meantime, adverse lipid levels serve as a major risk factor of cardiovascular diseases (9). Relations with those multiple complications make hyperlipidemia an unignorable risk for human health (10). Therefore, careful supervisory control and treatment of hyperlipidemia is a crucial issue for maintaining relative health of patients who were diagnosed (2).

Pre-diabetes is a condition between normal plasma glucose and type 2 diabetes (T2DM). Pre-diabetes was defined by impaired glucose tolerance (IGT) or impaired fasting glucose (IFG), and the global prevalence of IGT in 2021 was 9.1%, while IFG in 2021 was 5.8% (11). The prevalence of pre-diabetes among Chinese adult residents was 35.2%–38.1% (12, 13), and the rates of awareness and treatment of pre-diabetes are low in China (14). The all-cause mortality in pre-diabetic patients is 1.13 times higher than that in people without diabetes (15). Patients diagnosed with pre-diabetes are at higher risk of developing T2DM (16), and macrovascular complications may happen well before official diagnosis of diabetes (17). Management of lifestyle, weight, blood sugar, and blood lipids is essential for preventing the incidence of adverse events (18–23). On the aspect of blood lipids, individuals who are under condition of pre-diabetes tend to have higher prevalence of dyslipidemia than people whose plasma glucose are in the normal range; also, research has shown a positive association between high level of LDL-C and increased probability of pre-diabetes (24, 25). On the other hand, excess accumulation of cholesterol impaired glucose tolerance (26), which may accelerate the transition to diabetes in pre-diabetic patients. Therefore, timely identification and intervention of hyperlipidemia in pre-diabetes is important for keeping detrimental complications away from pre-diabetic patients (27).

HO-1 is an inducible form of heme oxygenase, a rate-limiting enzyme that degrades heme into biliverdin, carbon monoxide (CO), and ferrous iron (28) It exhibits a strong reaction to a wide range of chemical stress factors such as pro-inflammatory cytokines (29, 30) and serves as a protective molecule to defend against reactive oxygen species (ROS) (31). In recent years, accumulating studies suggested that HO-1 acted as a positive factor in numerous pathophysiological conditions. In terms of atherosclerotic lesions, the antioxidant, anti-inflammation, and anti-apoptotic functions of HO-1 play protective roles in the process (32, 33). There are also studies showing that HO-1 can mediate the reduction in ROS and low-density lipoprotein cholesterol (LDL-C) in some diabetes models (34, 35). Under the state of obesity in animal models, HO-1 starts to be upregulated to resist oxidant attack (36). Meanwhile, in the rodent model of obesity, insulin resistance and dyslipidemia can be rescued when HO-1 in adipocytes was increased (37). In addition, numerous clinical studies demonstrated the correlation between the expression level of HO-1 or heme degradants and obesity (28, 38).

Hyperlipidemia has strong connections with obesity, and it tends to induce metabolic inflammation in human body (39), whereas HO-1 is a protective factor against obesity and inflammation (28). Thus, we wonder if there exists a link between HO-1 and hyperlipidemia in patients with pre-diabetes. In this article, we detected the level of serum HO-1 in pre-diabetic patients and unveil the association between HO-1 and hyperlipidemia, providing a new insight of early detection and potential therapeutic targets in patients with morbid lipid metabolism.

## Method

### Study population and design

**Figure 1:**
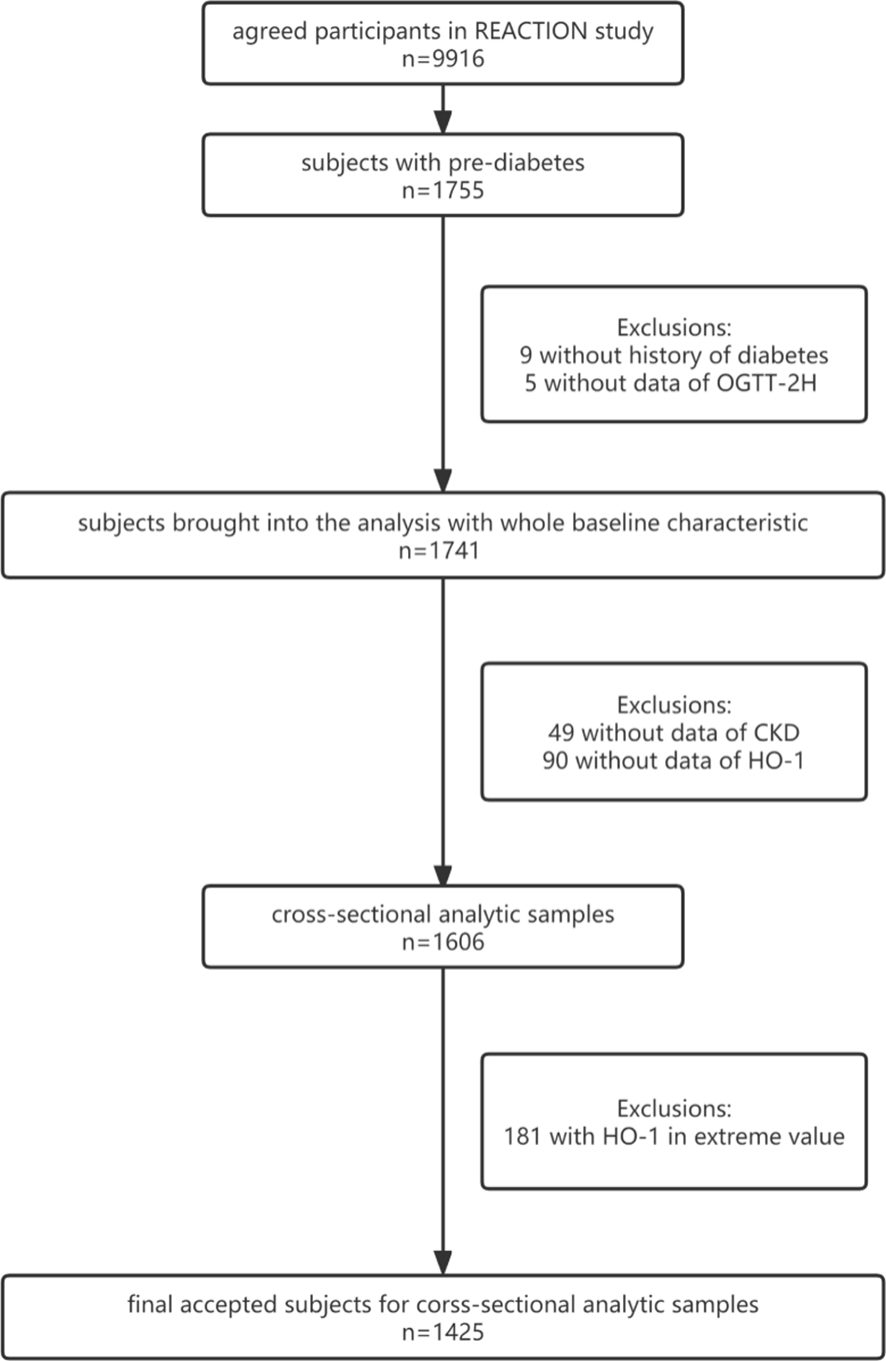
Patients screen flow chart.

Data were collected from patients in Sun Yat-sen Memorial Hospital, Sun Yat-sen University, Guangzhou, China, from June to November in 2011. The participants were also derived from REACTION study, which was designed as a multicenter Risk Evaluation of cAncers in Chinese diabeTic Individuals: A lONgitudinal (REACTION) prospective observational study (40). Within the REACTION study, we conveyed invitations to 10,104 residents through examination notices or home visits, among which 9,916 of them agreed to participate in the survey. We collected all the consent forms that the subjects signed. A total of 1,755 subjects with pre-diabetes were picked out based on the diagnosis criteria of diabetes on ADA 2010 (41). After exclusion of subjects who lacked history of diabetes and data of 2 h oral glucose tolerance test (OGTT-2H), we brought 1,741 subjects with baseline characteristics in 2011 into the cross-sectional analysis. Moreover, 49 subjects whose data on chronic kidney disease (CKD) were missing and 90 subjects without data of HO-1 were ruled out. At the same time, four subjects were also excluded for their missed data on both CKD and HO-1. In addition, we excluded 181 subjects with HO-1 in extreme value. In the end, 1,425 subjects who were not using hypoglycemic drugs were accepted into the analysis. Among the 1,425 subjects that we enrolled, 29.98% were men and 71.02% were women. The average age was 56.61 ± 7.30. Based on the definition of hyperlipidemia mentioned above, 176 subjects were diagnosed with hyperlipidemia and 1,249 subjects were not. The details of the screening process can be seen in **Figure 1**.

### Clinical and biochemical measurements

Anthropometric assessments were performed by well-trained staffs, and we used a standard questionnaire to record information on age, gender, and lifestyle including smoking and drinking habits. The category of smoking and drinking behaviors includes “yes” (individuals who kept smoking of drinking regularly in the last 6 months) and “no” (having quitted smoking or drinking for more than 6 months). Measurement of body weight and height took place with subjects wearing light clothes and without their shoes. The definition of body mass index (BMI) is weight in kilograms divided by height in meters squared (kg/m^2^). Before blood pressure measurement, subjects must refrain from consuming coffee, exercising, and smoking for at least 30 min. During the measurement, the bodies of the subjects were relaxed, and movement and conversation were avoided. Three measurements of the patient’s blood pressure were taken to calculate the average, and then, the results were recorded.

Participants were required to stay fasting overnight for at least 8 h before venous blood samples for laboratory test were collected. During laboratory tests, whole blood samples were collected from the vena cava and stored at −80°C. Indexes collected included TC, HDL-C, LDL-C, TG, fasting plasm glucose (FPG), hemoglobin A1C (HbA1C), aspartate aminotransferase (AST), alanine aminotransferase (ALT), and gamma-glutamyl transferase (GGT). All venous blood samples were tested by an autoanalyzer (Beckman CX-7 Biochemical Autoanalyzer, Brea, CA, USA). Homeostasis model assessments of insulin resistance (HOMA-IR) were determined based on the following simplified equations as first put forward by Turner in 1985: HOMA-IR = (FPI × FINS)/22.5. The results of venous blood samples for laboratory test were recorded by well-trained staffs.

### Definition of prediabetes, hyperlipidemia, and measurement of HO-1

According to the World Health Organization (WHO), pre-diabetes can be defined as follows: 1) isolated impaired glucose tolerance (IGT), FPG <7.0 mmol/L or OGTT-2H ≥7.8 to <11.1 mmol/L; 2) isolated impaired fasting glucose (IFG), FPG 6.1–6.9 mmol/L or OGTT-2H <7.8 mmol/L; and 3) combination of IGT and IFG (16, 42). Hyperlipidemia in our analysis was defined according to levels of TC > 5.17 mmol/L (>200 mg/dl) and TG > 2.26 mmol/L (200 mg/dl) (43). Blood samples from subjects were tested, and the level of serum HO-1 was quantitated by the commercially available ELISA kit (ab207621, Human Heme Oxygenase 1 SimpleStep ELISA^®^ Kit HO-1, USA) with an ultra-microvolume spectrophotometer (Thermo Fisher Scientific, USA).The ELISA kit recognizes human HO-1 protein and employs capture antibodies conjugated to an affinity tag that is recognized by the monoclonal antibody used to coat abcam SimpleStep ELISA^®^ plates of quantitate HO-1 with 7.9 pg/ml sensitivity.

### Statistical analysis

Data were analyzed with the statistical packages R (The R Foundation; http://www.r-project.org, version 4.2.0) and EmpowerStats (www.empowerstats.net, X&Y solutions, Inc. Boston, MA). Continuous variables were demonstrated by means ± standard deviation (SD). The ratios of categorical variables were presented. Data that were skewed continuous variables were analyzed with Kruskal–Wallis test to figure out the differences among groups, while χ^2^ test was used when data were categorical variables. Aimed to accurately distinguish the influence range of HO-1 on hyperlipidemia, we divided HO-1 into three groups as low [(0,45.20) pg/mL], middle [(45.20,210.18) pg/mL], and high level [(210.18,1746.18) pg/mL]. After stratified by sex, age, BMI, smoking habit, and drinking habit, we used logistic regression to explore the correlation between HO-1 and hyperlipidemia. Considering the potential differences in hyperlipidemia between genders, we further conducted logistic regression analysis after separating the data by gender based on weight and age. For a further investigation on the correlation between HO-1 and hyperlipidemia risk, multivariable analyses were performed after adjusted for sex, age, smoking, drinking, BMI, etc. To ensure the robustness of the regression model’s statistical results, we utilized propensity score matching (PSM) to matched the age and gender of the two groups with and without hyperlipidemia. The PSM included 1:1 greedy algorithm without replacement for the two groups, with a caliper width equal to 0.05. Additionally, we employed a genetic matching (GenMatch) algorithm to match the age and gender of the two groups for sensitivity analyses. Subsequently, multivariable analysis was conducted on the matched data sets to assess the correlation between HO-1 and hyperlipidemia after adjusting for covariates such as sex, age, smoking, drinking, and BMI. A two-tailed p < 0.05 was regarded to be statistically significant in all analyses.

## Result

### Clinical characteristics of subjects with or without hyperlipidemia

The biochemical and demographic characteristics of subjects in relation to hyperlipidemia are provided in **Table 1**. As shown in the biochemical test, the level of HO-1 was obviously decreased in hyperlipidemia subjects. In addition, the levels of LDL-C, OGTT-2H, HbA1C, AST, ALT, GGT, and HOMA-IR were significantly elevated except for HDL-C, which was downregulated (all p < 0.05). Compared with subjects without hyperlipidemia, the participants with hyperlipidemia tend to be older, heavier, and had higher BMI (all p < 0.05). Meanwhile, the proportions of female and non-smoker subjects were both higher compared with their counterparts within participants with hyperlipidemia. After conducting PSM matching based on participants’ age and gender, 176 subjects without hyperlipidemia and 176 subjects with hyperlipidemia were selected. The results indicated that the levels of HO-1 in subjects with hyperlipidemia were lower than those without hyperlipidemia (**Supplementary Table S1**). Using GenMatch for age and gender matching on the original data, the GenMatch matching algorithm generated more successful matches during the matching process to reduce bias between the two groups, thereby increasing the post-matching data volume. Consequently, after GenMatch matching, there were 11,013 subjects in both the group without hyperlipidemia and the group with hyperlipidemia, and the levels of HO-1 in the hyperlipidemia group remained lower than those in the group without hyperlipidemia (**Supplementary Table S2**).

**Table 1:** Characteristics stratified by hyperlipidemia.

	Hyperlipidemia		p-value
	No	Yes	
N	1249 (87.65%)	176 (12.35%)	
HO-1 (pg/ml)	322.95 ± 456.37	181.72 ± 309.57	<0.001
AGE (years)	56.39 ± 7.35	58.17 ± 6.75	0.002
Weight (kg)	59.96 ± 9.59	61.93 ± 9.11	0.011
BMI (kg/m^2^)	23.92 ± 3.08	24.60 ± 2.70	0.006
TC (mmol/L)	5.16 ± 1.18	6.42 ± 0.89	<0.001
TG (mmol/L)	1.48 ± 0.75	3.94 ± 2.34	<0.001
SBP (mmHG)	128.01 ± 15.85	133.63 ± 16.00	<0.001
HDL-C (mmol/L)	1.30 ± 0.35	1.21 ± 0.22	<0.001
LDL-C (mmol/L)	3.18 ± 0.94	3.55 ± 0.78	<0.001
OGTT2H (mmol/L)	8.68 ± 1.23	8.90 ± 1.20	0.027
HbA1C (mmol/L)	5.92 ± 0.43	6.01 ± 0.40	0.017
FPG (mmol/L)	5.66 ± 0.61	5.66 ± 0.60	0.916
AST (U/L)	19.09 ± 6.53	21.70 ± 6.22	<0.001
ALT (U/L)	15.06 ± 8.61	18.72 ± 10.67	<0.001
GGT (U/L)	24.34 ± 17.80	39.00 ± 26.53	<0.001
HOMA_IR	2.19 ± 1.27	2.72 ± 1.06	<0.001
Gender			0.021
Male	349 (27.94%)	64 (36.36%)	
Female	900 (72.06%)	112 (63.64%)	
SMOKE			0.016
No	1111 (91.22%)	147 (85.47%)	
Yes	107 (8.78%)	25 (14.53%)	
Alcohol			0.115
No	938 (76.95%)	120 (71.43%)	
Yes	281 (23.05%)	48 (28.57%)	

HO-1, heme oxygenase-1; BMI, body mass index; HDL-C, high-density lipoprotein cholesterol; LDL-C,low-density lipoprotein cholesterol; OGTT2H, 2 h oral glucose tolerance test; HbA1C, hemoglobin A1C; FPG, fasting plasm glucose; AST, aspartate aminotransferase; ALT, alanine aminotransferase; GGT, gamma-glutamyl transferase; HOMA-IR, homeostasis model assessment of insulin resistance. All data are reported as mean ± SD and absolute frequence where proper. p-value < 0.05 indicates significant.

### Association of HO-1 and hyperlipidemia in different adjusted models

**Figure 2:**
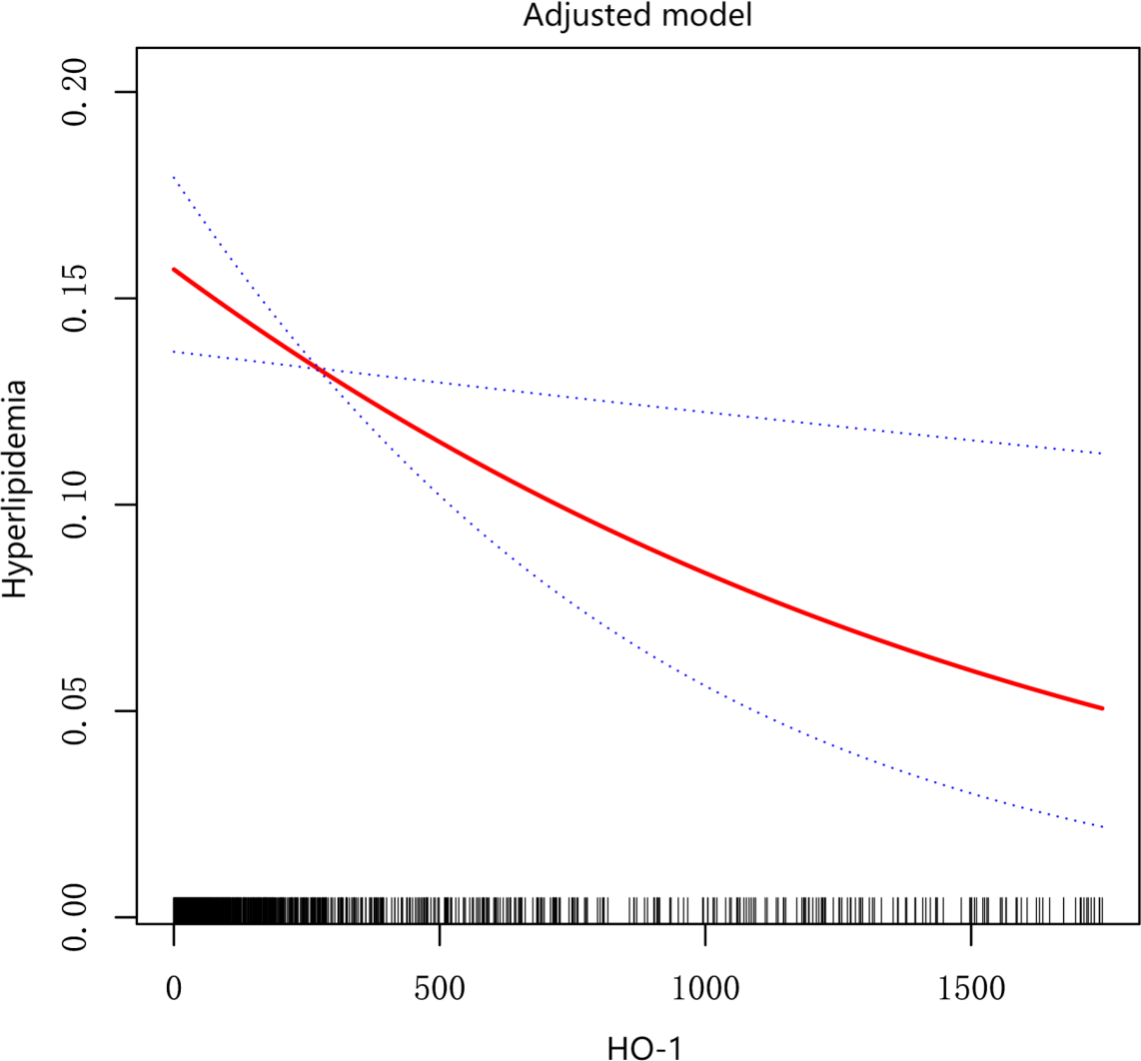
Association between HO-1 and hyperlipidemia after being adjusted.

To investigate whether HO-1 was an independent factor of hyperlipidemia, we incrementally adjusted confounding factors in five models as shown in **Table 2**. In model 1, without any adjustment of confounding factors, we found that high level of HO-1 correlated with reduced incidence of hyperlipidemia (OR, 0.46; 95% CI, 0.31–0.70, p = 0.0003), while no significant association between hyperlipidemia and low or middle level of HO-1. After adjusted by gender, age, smoke, alcohol, BMI, weight, SBP, LDL-C, FPG, AST, ALT, GGT, OGTT-2H, and HOMA-IR in model 5, we found that a high level of HO-1 had association with low incidence of hyperlipidemia [OR, 0.60; 95% CI, 0.37–0.97; p = 0.0367). As shown in **Figure 2**, after adjusting every confounding factor as model 5, we found that as the level of HO-1 rose, the incidence of hyperlipidemia decreased. To further confirm the correlation between HO-1 and hyperlipidemia, we conducted a similar analysis on the matched data mentioned above. In model 5, we still observed a negative correlation between a high level of HO-1 and hyperlipidemia (OR, 0.18; 95% CI, 0.06–0.52; p = 0.0015; and OR, 0.71; 95% CI, 0.66–0.77; p < 0.0001) (**Supplementary Tables S3**, **S4**).

**Table 2:** Association between HO-1 and hyperlipidemia in different adjusted models.

Models	n	Hyperlipidemia		
		Groups	OR (95% CI)	p-value
Model 1	1,425	Low	1	
		Middle	0.82 (0.57, 1.17)	0.2697
		High	0.46 (0.31, 0.70)	0.0003
Model 2	1,425	Low	1	
		Middle	0.80 (0.55, 1.15)	0.2256
		High	0.45 (0.29, 0.68)	0.0002
Model 3	1,365	Low	1	
		Middle	0.78 (0.54, 1.14)	0.1968
		High	0.43 (0.28, 0.66)	0.0001
Model 4	1,342	Low	1	
		Middle	0.84 (0.57, 1.23)	0.3718
		High	0.47 (0.31, 0.73)	0.0008
Model 5	1,196	Low	1	
		Middle	0.95 (0.63, 1.43)	0.8043
		High	0.60 (0.37, 0.97)	0.0367

Model 1: not adjusted.

Model 2: adjusted for gender, age.

Model 3: adjusted for gender, age, smoke, alcohol.

Model 4: adjusted for gender, age, smoke, alcohol, BMI, weight, SBP.

Model 5: adjusted for gender, age, smoke, alcohol, BMI, weight, SBP, LDL-C, FPG, AST, ALT, GGT, OGTT-2H, and HOMA-IR.

### Association of HO-1 and hyperlipidemia in different subgroups

To further explored which kinds of individuals had higher association between HO-1 and hyperlipidemia, we stratified subjects into five groups by gender, age, BMI, smoking habit, and drinking habit after adjusting every confounding factors. As shown in **Table 3**, a significant relationship between high level of HO-1 and hyperlipidemia was only detected in BMI between 24 and 30, which was defined as overweight (OR, 0.5; 95% CI, 0.3–0.9; p = 0.034). After stratifying age and BMI subgroups, respectively, by gender and adjusting potential confounding factors, we found a significant negative correlation between high levels of HO-1 and hyperlipidemia in the overweight group within female subjects, while for male participants, whether in the normal weight or overweight subgroup, no statistically significant relationship between HO-1 and hyperlipidemia was observed (OR, 0.4; 95% CI, 0.21–0.84; p = 0.014). Moreover, there was no statistically significant correlation between HO-1 and hyperlipidemia in subgroup of age regardless of gender (**Supplementary Table S5**).

**Table 3:** Effect of HO-1 on hyperlipidemia in different subgroup after adjusted potential confounding factors.

	N	Hyperlipidemia				
		Low	Middle	p-value	High	p-value
Gender						
Male	413	1	1.8 (0.9, 3.7)	0.118	0.5 (0.2, 1.1)	0.078
Female	1,012	1	0.7 (0.4, 1.2)	0.191	0.8 (0.4, 1.3)	0.342
Smoke						
No	1,258	1	0.8 (0.5, 1.2)	0.331	0.7 (0.4, 1.1)	0.099
Yes	132	1	3.7 (0.9, 15.4)	0.067	0.4 (0.1, 2.3)	0.310
Alcohol						
No	1,058	1	0.8 (0.5, 1.2)	0.271	0.6 (0.3, 1.0)	0.059
Yes	329	1	1.8 (0.8, 4.3)	0.157	0.8 (0.3, 2.1)	0.612
Age						
≤60	1,049	1	0.9 (0.6, 1.5)	0.801	0.6 (0.3, 1.0)	0.064
>60	376	1	1.1 (0.5, 2.5)	0.812	0.7 (0.3, 1.7)	0.407
BMI						
>18, ≤24	706	1	1.6 (0.8, 3.3)	0.198	1.0 (0.5, 2.2)	0.965
>24, ≤30	626	1	0.7 (0.4, 1.3)	0.263	0.5 (0.3, 0.9)	0.034*

All data were adjusted for gender, age, smoke, alcohol, BMI, weight, SBP, LDL-C, FPG, AST, ALT, GGT, OGTT-2H, and HOMA-IR. All data are shown in OR (95% confidence interval). A p-value of <0.05 indicates significance. *p < 0.05.

## Discussion

HO-1, as an anti-oxidative and anti-inflammation factor, has been proven to have lipid metabolic benefit in diabetic and non-alcoholic fatty liver disease (NAFLD) rodent model (34, 44–46). However few studies focus on effect of HO-1 on pre-diabetic patients who also had higher risk of hyperlipidemia than normal people. There is a lack of clinical evidence supporting the role of HO-1 in the treatment and prediction of hyperlipidemia on pre-diabetic patients.

In this study, we analyzed the association of HO-1 and hyperlipidemia in pre-diabetic patients. We discovered that a high level of HO-1 was negatively associated with risk of hyperlipidemia. Regression analysis on the matched data was conducted to confirm the stability of the original data analysis. Furthermore, we stratified pre-diabetic patients into different subgroups and found that a high level of HO-1 was mainly negatively associated with hyperlipidemia in overweight (BMI, 24–30) pre-diabetic patients, especially in female subjects. The has been a lot of experimental evidence resembling our results and those studies that proved that pharmacological or genetic induction of HO-1 in rodent models of obesity and diabetes decreased body weight and fasting blood glucose (10, 21–23). In addition, the incidence of non-alcoholic fatty liver disease (NAFLD) tends to decrease when the plasma bilirubin levels are high (47). According to the therapeutic potential of HO-1, we inferred that endogenous high level of HO-1 may resist to oxidative metabolic disorder just as our results show.

Here, we found that the level of HO-1 decreases in patients with hyperlipidemia. To our best knowledge, this article is the first one to look into the association of HO-1 and hyperlipidemia in pre-diabetic population. There are similar studies that found that the level of bilirubin, a consequent product of breakdown of heme, negatively correlates with BMI and obesity (48). In addition, it is found that the level of HO-1 decreases in patients with pre-diabetes and T2DM (49). Basing on this situation, we suppose that hyperlipidemia leads to a decrease in HO-1, as an increased ROS production can cause downregulation of HO-1 (50). However, our finding are surprisingly different from other studies claiming that elevated HO-1 level is associated with type 2 diabetes (51) and acute kidney injury (52).

In this study, we also observed significant abnormal changes in multiple physiological indicators in individuals with hyperlipidemia. First, there is a noticeable trend towards older age distribution in hyperlipidemia patients, indicating that older people have higher risk of developing hyperlipidemia as in previous studies (53, 54). Second, due to obesity being a significant factor in hyperlipidemia, patients with hyperlipidemia exhibit significantly higher body weight and BMI (55). Since the participants in this study were all prediabetic patients, their HOMA-IR levels were abnormally elevated. However, it was found that patients with hyperlipidemia had higher HOMA-IR levels compared to participants without hyperlipidemia. This is because insulin resistance is one of the risks of hyperlipidemia and is positively correlated with its occurrence (56). Additionally, liver function indicators such as AST, ALT, and GGT were significantly elevated in hyperlipidemia patients, possibly reflecting fatty liver and liver dysfunction (57). This further suggests a close association between hyperlipidemia and metabolic disorders, cardiovascular diseases, and conditions like fatty liver, providing important clues for further research on the pathogenesis and clinical interventions of hyperlipidemia.

The mechanism underlying the association between HO-1 and hyperlipidemia may attribute to the anti-oxidative and anti-inflammation capacity of HO-1. HO-1 and its antioxidants can increase formation of lipid metabolites like epoxyeicosatrienoic acid (EET) and 12,13-dihydroxy-9Z-octadecenoic acid (12,13-DiHOME), which attenuate the metabolic syndrome (58, 59). It is also found that the administration of EET leads to reduced fatty acid accumulation and improves the status of NAFLD in db/db mice (60), while inhibition of the antioxidant activity of HO exacerbates hepatic steatosis and fibrosis in hepatic cells (61). Studies also proved that HO-1 alleviates oxidative stress and reverses the adipocyte phenotype and hepatic steatosis by upregulating SIRT-1 and PPARα (62). In addition, in diet-induced obese mice with NAFLD, treatment of bilirubin nanoparticles decreased *de novo* lipogenesis and increased fat-burning β-oxidation (63). Due to the above features of HO-1, a relatively high level of this oxygenase is possibly beneficial to the incidence of hyperlipidemia.

Under status of pre-diabetes, the chances of developing dyslipidemia and T2DM (24) are high and the level of HO-1 decreases (49). Although the association of HO-1 and diabetes is specific, few studies look into the relationship of hyperlipidemia and HO-1 in pre-diabetic patients. Our study replenishes the void in clinical proof of the relationship between HO-1 and hyperlipidemia in pre-diabetes. In the current study, we also found that the level of HO-1 is negatively related to overweight subjects especially female subjects with hyperlipidemia. This indicates that high levels of HO-1 are inversely related to hyperlipidemia and can be influenced by weight and gender. HO-1 is a crucial antioxidant enzyme known for its antioxidative, anti-inflammatory, and cell protective functions as mentioned. Women generally have higher body fat compared to men due to lower basal metabolic rates and muscle content (64, 65). In overweight female patients, excessive weight and female hormone levels may worsen oxidative stress and inflammation, increasing the risk of hyperlipidemia (66). Reduced levels of HO-1 could lead to higher oxidative stress and inflammation, worsening lipid metabolism disorders, and hyperlipidemia development (67). Therefore, in overweight female patients, the anti-oxidative and anti-inflammatory effects of HO-1 may negatively regulate the occurrence of hyperlipidemia, showing an inverse correlation. However, this hypothesis requires further investigation through expanding sample size or conducting basic research using animal models.

### Limitation

Several limitations exist in our study. The number of samples may need to expand to provide solid proof of our conclusion. Moreover, we only investigated Chinese patients, so whether the results can be generalized to patients from other areas is not clear. In this article, we discuss the association of endogenous HO-1 and hyperlipidemia, so the results can only serve as a hint on the potency of HO-1 treatment but not a direct proof on HO-1 medical capacity in human flesh. Although we draw a conclusion that high level of HO-1 associates with reduced possibility of hyperlipidemia, how to maintain the level of HO-1 in human body is elusive. Due to the lack of data on newly diagnosed diabetes patients and those with long-term diabetes, this study was unable to compare between pre-diabetic patients and diabetic patients.

## Conclusions

A high level of HO-1 was negatively associated with hyperlipidemia in pre-diabetic patients especially in overweight female ones. This study provide information on the exploratory study of the mechanism of HO-1 in hyperlipidemia while also suggesting that its mechanism may be influenced by body weight and gender.

## Data availability statement

The original contributions presented in the study are included in the article/**Supplementary Material**. Further inquiries can be directed to the corresponding author.

## Ethics statement

The studies involving humans were approved by Ethics Committee of Sun Yat-sen Memorial Hospital, Sun Yat-sen University. Ethics Approval Number: Sun Yat-Sen Memorial Hospital of Zhongshan University (2019) Ethical Approval Research No. (38). The studies were conducted in accordance with the local legislation and institutional requirements. The participants provided their written informed consent to participate in this study. Written informed consent was obtained from the individual(s) for the publication of any potentially identifiable images or data included in this article.

## Author contributions

SF: Writing – original draft, Writing – review & editing. YY: Writing – original draft, Writing – review & editing. XL: Data curation, Methodology, Writing – review & editing. JL: Data curation, Methodology, Writing – review & editing. YQ: Data curation, Writing – review & editing. LY: Funding acquisition, Supervision, Writing – review & editing. MR: Supervision, Visualization, Writing – original draft, Writing – review & editing.
