# Supplementary material for: Association between heme oxygenase-1 and hyperlipidemia in pre-diabetic patients: a cross-sectional study
Source: Front Endocrinol (Lausanne). 2024 May 23;15:1380163. doi: 10.3389/fendo.2024.1380163 (PMC11153693; doi:10.3389/fendo.2024.1380163)
Supplement: Supplementary file 1 [file Table_1.docx]

**Supplement table 1 Characteristics stratified by hyperlipidemia after usin PSM matching.**

|  | Hyperlipidemia-after PSM matching | | P-value |
| --- | --- | --- | --- |
|  | No | Yes |  |
| N | 176 (50%) | 176 (50%) |  |
| HO-1(pg/mL) | 274.12 ± 448.00 | 181.72 ± 309.57 | 0.025 |
| AGE (years) | 48.18 ± 6.61 | 58.17 ± 6.75 | <0.0001 |
| WEIGHT (kg) | 60.13 ± 9.22 | 61.93 ± 9.11 | 0.0684 |
| BMI (kg/m2) | 24.05 ± 2.90 | 24.60 ± 2.70 | 0.0679 |
| TC (mmol/L) | 5.01 ± 1.07 | 6.42 ± 0.89 | <0.0001 |
| TG (mmol/L) | 1.46 ± 0.68 | 3.94 ± 2.34 | <0.0001 |
| SBP (mmHG) | 123.70 ± 13.37 | 133.63 ± 16.00 | <0.001 |
| HDL-C (mmol/L) | 1.30 ± 0.34 | 1.21 ± 0.22 | 0.0034 |
| LDL-C (mmol/L) | 3.07 ± 0.88 | 3.55 ± 0.78 | <0.0001 |
| OGTT2H (mmol/L) | 8.53 ± 1.23 | 8.90 ± 1.20 | 0.0048 |
| HbA1C (mmol/L) | 5.81 ± 0.47 | 6.01 ± 0.40 | <0.0001 |
| FPG (mmol/L) | 5.56 ± 0.61 | 5.66 ± 0.60 | 0.136 |
| AST (U/L) | 17.35 ± 5.50 | 21.70 ± 6.22 | <0.0001 |
| ALT (U/L) | 13.95 ± 7.03 | 18.72 ± 10.67 | <0.0001 |
| GGT (U/L) | 21.61 ± 14.38 | 39.00 ± 26.53 | <0.0001 |
| HOMA_IR | 2.22 ± 1.09 | 2.72 ± 1.06 | <0.0001 |
| Gender |  |  | <0.0001 |
| male | 18 (10.2) | 64 (36.4) |  |
| female | 158 (89.8) | 112 (63.6) |  |
| SMOKE |  |  | 0.0001 |
| No | 167 (97.7) | 147 (85.5) |  |
| Yes | 4 (2.3) | 25 (14.5) |  |
| ALCOHOL |  |  | 0.1879 |
| No | 133 (78.2) | 120 (71.4) |  |
| Yes | 37 (21.8) | 48 (28.6) |  |

HO-1：heme oxygenase-1, BMI: body mass index, HDL-C: high-density lipoprotein cholesterol, LDL-C: low-density lipoprotein cholesterol, OGTT2H: two hours oral glucose tolerance test, HbA1C: hemoglobin A1C, FPG: fasting plasm glucose, AST: aspartate aminotransferase, ALT: alanine aminotransferase, GGT: gamma-glutamyl transferase, HOMA_IR: homeostasis model assessment of insulin resistance. All data are reported as mean ± SD and absolute frequence where proper. After PSM matching group used PSM propensity scores for matching. P value < 0.05 indicates significant.

**Supplement table 2 Characteristics stratified by hyperlipidemia after usin GenMacth matching.**

|  | Hyperlipidemia-after GenMatch matching | | P-value |
| --- | --- | --- | --- |
|  | No | Yes |  |
| N | 11013 (50%) | 11013 (50%) |  |
| HO-1(pg/mL) | (11013) 295.11 ± 438.42 | (11013) 173.58 ± 306.69 | <0.0001 |
| AGE (years) | (11013) 56.50 ± 4.56 | (11013) 56.50 ± 4.51 | 0.9727 |
| WEIGHT (kg) | (10872) 58.61 ± 9.16 | (10679) 60.29 ± 8.16 | <0.0001 |
| BMI (kg/m2) | (10840) 23.81 ± 3.13 | (10667) 24.57 ± 2.78 | <0.0001 |
| TC (mmol/L) | (11013) 5.27 ± 1.23 | (11013) 6.45 ± 0.88 | <0.0001 |
| TG (mmol/L) | (10983) 1.47 ± 0.70 | (11013) 3.94 ± 2.36 | <0.0001 |
| SBP (mmHG) | (10827) 127.17 ± 15.63 | (10881) 132.31 ± 15.82 | <0.0001 |
| HDL-C (mmol/L) | (11013) 1.34 ± 0.36 | (11013) 1.24 ± 0.22 | <0.0001 |
| LDL-C (mmol/L) | (11011) 3.23 ± 0.95 | (11013) 3.56 ± 0.82 | <0.0001 |
| OGTT2H (mmol/L) | (11013) 8.69 ± 1.20 | (11013) 8.90 ± 1.12 | <0.0001 |
| HbA1C (mmol/L) | (11013) 5.95 ± 0.42 | (11013) 6.03 ± 0.37 | <0.0001 |
| FPG (mmol/L) | (11013) 5.68 ± 0.60 | (11013) 5.67 ± 0.59 | 0.2075 |
| AST (U/L) | (11007) 19.22 ± 6.77 | (11013) 21.94 ± 6.33 | <0.0001 |
| ALT (U/L) | (9732) 14.91 ± 8.60 | (10833) 18.86 ± 10.04 | <0.0001 |
| GGT (U/L) | (10985) 23.48 ± 17.57 | (11013) 36.63 ± 24.53 | <0.0001 |
| HOMA_IR | (11013) 2.22 ± 1.35 | (11013) 2.77 ± 1.08 | <0.0001 |
| Gender |  |  | 1 |
| male | 1644 (14.9) | 1645 (14.9) |  |
| female | 9369 (85.1) | 9368 (85.1) |  |
| SMOKE |  |  | <0.0001 |
| No | 10245 (95.3) | 10207 (93.2) |  |
| Yes | 502 (4.7) | 747 (6.8) |  |
| ALCOHOL |  |  | 0.0305 |
| No | 8746 (81.1) | 8401 (80) |  |
| Yes | 2032 (18.9) | 2105 (20) |  |

HO-1：heme oxygenase-1, BMI: body mass index, HDL-C: high-density lipoprotein cholesterol, LDL-C: low-density lipoprotein cholesterol, OGTT2H: two hours oral glucose tolerance test, HbA1C: hemoglobin A1C, FPG: fasting plasm glucose, AST: aspartate aminotransferase, ALT: alanine aminotransferase, GGT: gamma-glutamyl transferase, HOMA_IR: homeostasis model assessment of insulin resistance. All data are reported as mean ± SD and absolute frequence where proper. After PSM matching group used GenMatch propensity scores for matching. P value < 0.05 indicates significant.

**Supplement table 3 Association between HMOX1 and hyperlipidemia in different adjusted models before and after PSM matching.**

| models | n |  | Hyperlipidemia-after PSM maching | |
| --- | --- | --- | --- | --- |
|  |  | Groups | OR (95%CI) | P_value |
| Model 1 | 352 | Low | 1 |  |
|  |  | Middle | 0.87 (0.51, 1.46) | 0.5925 |
|  |  | High | 0.24 (0.14, 0.42) | <0.0001 |
| Model 2 | 352 | Low | 1 |  |
|  |  | Middle | 0.79 (0.40, 1.57) | 0.4997 |
|  |  | High | 0.17 (0.08, 0.36) | <0.0001 |
| Model 3 | 336 | Low | 1 |  |
|  |  | Middle | 0.81 (0.40, 1.65) | 0.5681 |
|  |  | High | 0.17 (0.08, 0.37) | <0.0001 |
| Model 4 | 332 | Low | 1 |  |
|  |  | Middle | 0.82 (0.40, 1.69) | 0.5956 |
|  |  | High | 0.19 (0.09, 0.41) | <0.0001 |
| Model 5 | 277 | Low | 1 |  |
|  |  | Middle | 0.67 (0.26, 1.73) | 0.4033 |
|  |  | High | 0.18 (0.06, 0.52) | 0.0015 |

Model 1: not adjusted

Modle 2: adjusted for Gender, Age

Modle 3: adjusted for Gender, Age, Smoke, Alcohol

Model 4: adjusted for Gender, Age, Smoke, Alcohol, BMI, Weight, SBP

Model 5: adjusted for Gender, Age, Smoke, Alcohol, BMI, Weight, SBP, LDL-C, FPG, AST, ALT, GGT, OGTT-2H and HOMA_IR

**Supplement table 4 Association between HMOX1 and hyperlipidemia in different adjusted models before and after GenMatch matching.**

| models | n |  | Hyperlipidemia-after GenMatch maching | |
| --- | --- | --- | --- | --- |
|  |  | Groups | OR (95%CI) | P_value |
| Model 1 | 352 | Low | 1 |  |
|  |  | Middle | 0.79 (0.74, 0.84) | <0.0001 |
|  |  | High | 0.49 (0.46, 0.52) | <0.0001 |
| Model 2 | 352 | Low | 1 |  |
|  |  | Middle | 0.78 (0.73, 0.84) | <0.0001 |
|  |  | High | 0.49 (0.45, 0.52) | <0.0001 |
| Model 3 | 336 | Low | 1 |  |
|  |  | Middle | 0.78 (0.73, 0.83) | <0.0001 |
|  |  | High | 0.50 (0.47, 0.54) | <0.0001 |
| Model 4 | 332 | Low | 1 |  |
|  |  | Middle | 0.86 (0.80, 0.92) | <0.0001 |
|  |  | High | 0.60 (0.56, 0.64) | <0.0001 |
| Model 5 | 277 | Low | 1 |  |
|  |  | Middle | 0.96 (0.89, 1.04) | 0.2863 |
|  |  | High | 0.71 (0.66, 0.77) | <0.0001 |

Model 1: not adjusted

Modle 2: adjusted for Gender, Age

Modle 3: adjusted for Gender, Age, Smoke, Alcohol

Model 4: adjusted for Gender, Age, Smoke, Alcohol, BMI, Weight, SBP

Model 5: adjusted for Gender, Age, Smoke, Alcohol, BMI, Weight, SBP, LDL-C, FPG, AST, ALT, GGT, OGTT-2H and HOMA_IR

**Supplement table 5 Association between HMOX1 and hyperlipidemia after adjusted of confounding factors by stratifying different age and BMI groups by gender**

|  |  |  | Hyperlipidemia | | | |  |
| --- | --- | --- | --- | --- | --- | --- | --- |
|  |  |  | Low | Middle | P_value | High | P_value |
|  |  | Age |  |  |  |  |  |
| Gender | Male | <=60 | 1 | 0.69 (0.28, 1.70) | 0.419 | 0.71 (0.31, 1.66) | 0.429 |
|  |  | >60 | 1 | 0.45 (0.14, 1.49) | 0.192 | 0.44 (0.14, 1.33) | 0.146 |
|  | Female | <=60 | 1 | 0.75 (0.47, 1.18) | 0.207 | 0.71 (0.44, 1.14) | 0.156 |
|  |  | >60 | 1 | 1.05 (0.36, 3.06) | 0.927 | 0.51 (0.18, 1.45) | 0.206 |
|  |  | BMI |  |  |  |  |  |
| Gender | Male | >18, <=24 | 1 | 0.95 (0.34, 2.65) | 0.917 | 0.91 (0.34, 2.43) | 0.846 |
|  |  | >24, <=30 | 1 | 0.43 (0.13, 1.38) | 0.156 | 0.41 (0.14, 1.24) | 0.115 |
|  | Female | >18, <=24 | 1 | 1.09 (0.61, 1.96) | 0.768 | 0.87 (0.48, 1.58) | 0.644 |
|  |  | >24, <=30 | 1 | 0.53 (0.28, 1.03) | 0.061 | 0.42 (0.21, 0.84) | 0.014* |

All data were adjusted for Age, Smoke, Alcohol, BMI, Weight, SBP, LDL-C, FPG, AST, ALT, GGT, OGTT-2H and HOMA_IR

All data are shown in OR (95% confidence interval). P value of < 0.05 indicates significance. *P < 0.05
